# Empyema of the Antrum

**Published:** 1893-12

**Authors:** A. B. D. Densham


ARTICLE II.

### EMPYEMA OF THE ANTRUM.

BY A. B. D. DENSHAM.

So much has been said and written about this condition, that it will perhaps be better to give merely the outlines of the methods of operative treatment.

The indications are to remove all sources of irritation, to evacuate pus, to maintain free drainage, and to prevent decomposition of secretions. The position in which the antrum should be opened varies according to circumstances. As a general rule all dead teeth and unhealthy roots on the affected side should be removed, and the socket through which pus escapes should be enlarged. Usually the socket of the first molar is chosen when none of the others communicate with the cavity, for this tooth is the most frequent cause of empyema, and it also occupies the most dependent position in the floor of the antrum. To enlarge the opening through this socket a large trocar or a small bone gauge is generally used, and with ordinary care no harm can be done with these instruments. As a considerable amount of force is necessary to perforate the bone, when this is accomplished the instrument is apt to slip further than is intended and may be driven against the roof of the antrum, or even perforate that bony process and wound the orbit, to guard against this untoward accident, the operator's index finger should be firmly held against the shaft of the naked trocar about three-quarters of an inch from the point, thus however suddenly the perforation is completed, the finger checks further progress of the point of the instrument.* The opening thus secured should be enlarged by rotatory movements of the trocar, or by a reamer. It is of the greatest importance to secure a free opening. An error that is frequently made in the treatment of these cases is the production of a small aperture only; this is insufficient for adequate drainage and a small opening will not allow of the passage of flakes of inspissated pus of any size, besides preventing the use of injections in sufficient volume to be of any value. Having in this manner opened the antrum and excavated a certain amount of pus, the cavity should be thoroughly flushed with a fairly forcible stream of antiseptic fluid, in order that any pus which may be collected in the dependent portions of the cavity, or in the loculi sometimes present

---

*Never use the instruments named if the dental, or so-called surgical engine is at hand.

in the floor, may be thoroughly removed. If a strong antiseptic has been used, the cavity must then be flushed with a weaker solution to prevent absorption. When empyema of the antrum is associated with necrosis of some portion of its bony wall, the pus evacuated has a very characteristic odor which is easily recognized by any one who has ever smelt pus in connection with dead bone. When this odor is detected the cavity of the antrum should be carefully explored with a probe in all directions in order to ascertain the position of the bare bone; if it is found that the dead bone is loose its removal should be effected by means of sequestrum forceps with long thin shanks, or by a sharp spoon with a thin shank which can be adapted to any required angle.

Another position in which the antrum may be opened is through the base of the molar process of the superior maxillary bone, above the alveolar process. This method is especially applicable where a larger opening is required than can be made through the socket of a tooth, where all the teeth on the effected side are sound, in edentulous cases where the alveolar process is very dense, and in cases where the removal of a foreign body is the aim of the operation. This may be affected by everting the cheek, incising the mucous membrane and exposing the bone above the position of the second molar tooth, and then perforating here with a gimblet, a stout trocar, or a pair of strong scissors held closed in the hand and bored into the bone with a rotatory movement.* By this method it is sometimes difficult to tell exactly when the cavity of the antrum is reached; it is therefore necessary to withdraw the instrument occasionally and ascertain ths state of affairs by means of a probe; further procedures are as before described.

When the operation is undertaken for the removal of a foreign body, the antrum may be opened in the way de-

*Primitive surgery indeed.

scribed and the effect of injection of a stream of warm water at a moderate pressure may be tried; if this fails to dislodge the offending body, the cavity may be thoroughly explored with forceps of various shapes, or a rubber cup attached to a soft wire stem may be found very useful with the same intent. The subsequent treatment of these cases during the process of repair needs no mention in this paper.—*Dental Record.*

